# A scoping review of factors associated with premarital sex-related risky sexual health behavior among adolescents in conservative societies based on the theory of planned behavior

**DOI:** 10.1186/s12889-025-25665-x

**Published:** 2025-12-04

**Authors:** Jerwilsem Andrulin Latuheru, Iyus Yosep, Aat Sriati

**Affiliations:** 1 Master Study Program, Faculty of Nursing, Universitas Padjadjaran, Sumedang, West Java Indonesia; 2 Department of Mental Health Nursing, Faculty of Nursing, Universitas Padjadjaran, Jl. Proklamasi Km. 21, Jatinangor, Sumedang, Indonesia

**Keywords:** Adolescent, Premarital sex, Risky sexual behavior, Conservative societies, Theory of planned behavior

## Abstract

**Background:**

Premarital sexual behavior among adolescents remains a sensitive public health concern in conservative societies, where cultural and religious norms strictly regulate sexuality. Despite these limitations, risky behaviors persist and are influenced by environmental, familial, and media factors.

**Objective:**

This review aimed to identify and synthesize factors related to premarital sexual behavior among adolescents in conservative societies using the Theory of Planned Behavior (TPB) as a guiding framework.

**Methods:**

A scoping review was conducted according to The 2020 Preferred Reporting Items for Systematic Reviews and Meta-Analyses for Scoping Review (PRISMA-ScR) guidelines. Relevant studies were identified in PubMed, Scopus, Cochrane, and EBSCOhost, focusing on adolescents aged 10–24 years in conservative settings. The data were analyzed thematically based on the following TPB components: attitudes, subjective norms, and perceived behavioral control.

**Results:**

Twenty-three studies met our inclusion criteria. The factors influencing premarital sex include religiosity, peer pressure, exposure to pornography, parental supervision, and substance use. Religiosity and family communication act as protective factors, whereas exposure to pornography and peer influence increases risk.

**Conclusion:**

Premarital sexual behavior among adolescents in conservative societies is shaped by individual attitudes, social norms, and perceived control. Culturally sensitive sex education programs, digital literacy, and family involvement are recommended to promote safer behaviors.

**Supplementary Information:**

The online version contains supplementary material available at 10.1186/s12889-025-25665-x.

## Introduction

Premarital sex is defined as sexual intercourse among young people before the age of 25 years or between partners prior to legal marriage [1–3]. This activity includes various forms of physical intimacy outside marriage [2]. In many conservative societies such as Iran, Indonesia, and Malaysia, where the population is predominantly Muslim and upholds traditional values, sexual relations outside marriage are both religiously and legally forbidden. Religious and cultural norms strongly condemn premarital sexual relations and highly value virginity before marriage, particularly among women [4]. Conservative societies often view premarital sexual relations as violations of religious and cultural norms. However, to avoid premarital sexual relations, adolescents often engage in other risky behaviors, such as alcohol and drug use, or watch pornography as an alternative to expressing their sexual curiosity without violating social norms [5]. Premarital sexual activity generally occurs during adolescence, especially between the ages of 14 and 19 years, when young people are most vulnerable to engaging in such behavior [6, 7]. The prevalence of premarital sex increased in many countries between 1999 and 2018 [8]. For example, rates have been reported to be as high as 85% in Italy and as low as 10.1% in China [8], reflecting substantial variation across cultural contexts. Premarital sexual behavior is influenced not only by biological and cognitive development but also by psychosocial factors such as exposure to pornography, peer groups, and family dynamics [9].

Premarital sex, especially when unprotected or involving multiple partners, is associated with significant reproductive health risk [10]. Condom use among adolescents has recently declined: among boys, from 70% in 2014 to 61% in 2022, and among girls, from 63% to 57% during the same period [11]. Risks include sexually transmitted infections (STIs) such as HIV/AIDS, gonorrhea, and syphilis [12]. More than 50% of adolescents have limited knowledge about HIV, and nearly half hold negative attitudes toward HIV prevention [13]. Premarital sex also contributes to teenage pregnancy, which can cause serious complications for both mother and child. Beyond physical health, it may negatively affect adolescents’ mental health, leading to regret, loss of self-confidence, depression, and suicidal ideation in some cases [7, 14, 15]. The social stigma associated with premarital sex often exacerbates these psychological impacts through self-stigma and shame.

This review applies the Theory of Planned Behavior (TPB) as a guiding framework to better understand complex issues related to adolescent sexual and reproductive health. According to TPB, an individual’s intention to perform a behavior is shaped by three factors: attitude toward the behavior, subjective norms, and perceived behavioral control [16]. The interaction between attitude, subjective norms, and perceived behavioral control influences adolescents’ behavioral decisions. Attitude is influenced by experience and knowledge, and subjective norms are related to family and peer influence. Perceived behavioral control reflects how much an individual feels they have control over their behavior, influenced by external and internal factors, which can further shape the intention to perform a behavior. Although TPB has been widely applied in health behavior research, relatively few studies have comprehensively integrated multiple factors influencing adolescent premarital sexual behavior within this framework [17, 18]. Prior systematic reviews have mainly identified factors such as peer pressure, pornography exposure, smoking, and alcohol consumption [19, 20] but have paid less attention to other domains of attitudes and subjective norms. Therefore, this scoping review explores the factors associated with premarital sexual behavior among adolescents in conservative societies using the Theory of Planned Behavior as a framework.

## Methods

### Design

The study used a scoping review approach according to the Preferred Reporting Items for Systematic Review and Meta-analysis – Extended for Scoping Review (PRISMA-ScR).

### Eligibility criteria

The inclusion criterion for this study was the PCC framework (Population, Concept, and Context). Population: According to the World Health Organization (WHO), UNICEF, and the United Nations Population Fund (UNFPA), adolescence is formally defined as an age range of 10–19 years. UNFPA also recognizes the category of adolescents (15–24 years) and the broader term “young people” (10–24 years) [21]. Excluding these studies would result in the loss of significant amounts of relevant evidence. This review focuses on premarital sex, specifically referring to sexual relations among adolescents or couples before legal marriage. The context discussed is risky sexual behavior, defined as any sexual activity that increases the risk of sexually transmitted infections (STIs) or pregnancy.

Inclusion criteria: We included primary studies (quantitative, qualitative, or mixed methods) that explicitly examined factors related to premarital sexual behavior among young people under 25 years of age in conservative societies. In this review, the UNESCO definition of conservative societies states that in such societies, sexual relations outside marriage are typically considered forbidden due to strong cultural or religious beliefs. The norms governing sexuality are influenced by this moral code, which also affects health-related sexual behaviors [22]. Eligible studies were those published in English and available in full text without restrictions on the year of publication. Exclusion criteria: We excluded non-original research such as reviews, editorials, comments, and case reports; research without full-text access; studies that did not address premarital sex factors in adolescents; and studies whose populations were not within the specified age range (< 25 years). We also excluded studies that focused only on general risky sexual behavior without specifically analyzing premarital sexual behavior.

### Search strategy

The search was conducted on May 15, 2025, for relevant articles published in four databases: EBSCOhost, PubMed, Scopus, and Cochrane Central Register of Controlled Trials (CENTRAL). The search strategy used keywords tailored to *Medical Subject Headings* (MeSH) *terms*. The keywords include “*Adolescent OR Adolescents OR Teen OR Teens AND Premarital sex” OR “Premarital sexual intercourse AND Predictor OR Factors OR Determinant*”. Cochrane uses “*Adolescent OR Adolescents OR Teen OR Teens OR Youth OR Young AND premarital sex OR premarital sexual intercourse OR premarital sexual behavior*.” Supplementary searches were manually performed on reference compilations of potentially pertinent publications (see Supplementary File 1).

### Study selection and quality appraisal

The study selection process was conducted in stages according to the PRISMA 2020 guidelines. First, all search results were obtained from four databases (PubMed, Scopus, Cochrane Library, and EBSCOhost). Two authors (J.A.L. and S.R.) selected studies that met these criteria. Another author (I.Y.) checked for duplication in the initial selection process by using the Mendeley application. All authors (J.A.L., I.Y., and S.R.) reviewed the titles, abstracts, and full texts for relevance to the research topic and established the inclusion and exclusion criteria for the review. When several articles were based on the same dataset or research population, researchers retained only the most comprehensive or methodologically rigorous study to avoid duplication. Any differences in opinions between the two reviewers were resolved through discussion until a consensus was reached. No formal quality appraisal was applied, as the objective of this scoping review was to map existing evidence rather than to assess study quality [23].

### Data extraction and data analysis

Data extraction from the included studies was independently performed by three reviewers using a standardized extraction form. The information collected included the author’s name and year of publication, country of origin of the research, research design, sample size, demographic characteristics of the participants such as gender (percentage) and age (age category), and factors reported to be related to premarital sexual behavior. To avoid duplication of evidence, we carefully checked for potential overlap among the included studies by cross-examining dataset names, study settings, sample sizes, and recruitment periods. Only the most comprehensive or methodologically rigorous articles were retained for the analysis when multiple publications were derived from the same dataset or overlapping populations. The accompanying papers were reviewed for context, but not counted as separate studies, thus ensuring the independence of the estimates. Next, the identified factors were mapped and categorized based on domains within the Theory of Planned Behavior (TPB), namely, attitudes, subjective norms, and perceived behavioral control, to ensure consistency in interpretation. Any differences in the data extraction process were resolved through discussion until consensus was reached.

## Results

### Study selection

**Fig. 1:**
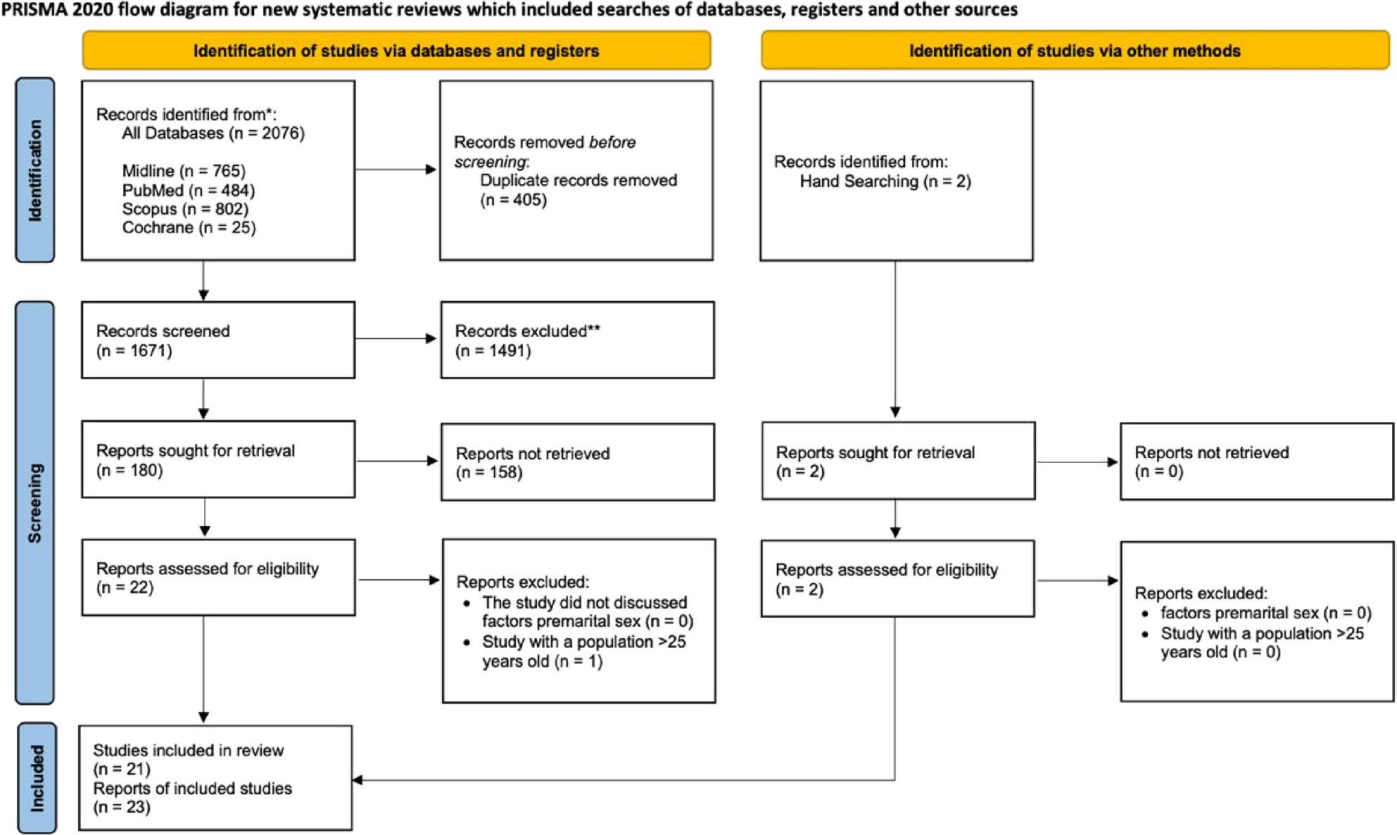
PRISMA 2020 Flow Diagram [44]

A total of 2,076 articles were identified using these four databases. After checking for duplicates, 405 duplicate articles were removed, and 1,671 articles were selected for further screening. A total of 1,491 articles were excluded because they did not meet the screening criteria. The title and abstract screening process yielded 22 articles that met the eligibility criteria for a full-text review. After further examination, one article was excluded because of irrelevant data or not being applicable to the population under twenty-five years old. Thus, a total of 21 articles were included in the analysis. Two additional articles were identified through manual searching. Finally, this review included 23 articles that met the inclusion criteria (Fig. 1) [5, 24–43]. This rigorous selection process ensured that the final selected articles aligned with the objectives of the scoping review on Factors Associated with Premarital Sex-Related Risky Sexual Health Behavior among Adolescents in Conservative Societies.

### Characteristics of included studies

The 23 studies included in this review covered publications published from 2009 to 2025. The majority of the analyzed studies were conducted in developing countries, particularly in Africa and Asia. The included countries were Ethiopia (*n* = 10) [24–32], Indonesia (*n* = 4) [33–36], Thailand (*n* = 2) [37, 38], Malaysia (*n* = 1) [5], Benin (*n* = 1) [39], Nepal (*n* = 1) [40], Africa (*n* = 1) [1], China (*n* = 1) [41], Singapore (*n* = 1) [42], and Nigeria (*n* = 1) [43]. 

(10–13 early, 14–16 middle, 17–19 late adolescence, and 20–24 youth) [21].

The study with the largest number of participants in this review included 87,924 adolescents aged 15–24, all of whom were female [1]. Conversely, the study with the smallest number of participants included 109 adolescents aged 15–18, with the majority of participants being male [33]. Additionally, all studies included in this review reported the prevalence of premarital sex (Table 1).

**Table 1:** Characteristics of study

Characteristic	Number of studies, *n*	Reference
Study design	**23**	[5, 24–43]
Quantitative Cross-Sectional	21	[5, 24, 25], 27– [41, 43]
Quantitative Case-Control	1	[42]
Mixed-method Cross-sectional	1	[26]
Country	**10**	
Ethiopia	10	[24–32]
Indonesia	4	[33–36]
Thailand	2	[33, 38]
Malaysia	1	[5]
Benin	1	[39]
Nepal	1	[40]
Africa (multi-country)	1	[1]
China	1	[41]
Singapore	1	[42]
Nigeria	1	[43]
Sample size (range)	109–87.924 adolescents	[5, 24–43]
Gender distribution		
Male and female (mixed)	14	[5, 24], 31– [34], 37– [40]
Female only	5	[1, 29, 30, 41, 43]
Male only	1	[35]
Unclear/not specified	2	[36, 42]
Age group (Adolescent stage)		
Early adolescence (10–13 years)	0	
Middle adolescence (14–16 years)	4	[30, 36, 37, 42]
Late adolescence (17–19 years)	6	[2, 5, 25, 32, 33, 38]
Youth (20–24 years)	13	[24], 26– [29, 31, 34, 35], 39– [41, 43]
Prevalence of premarital sex	4.2% – 69%	[5, 24–43]
Predictors of premarital sex		
Knowledge	6	[31, 32, 36], 38– [40]
Religion	6	[1, 5, 28, 31, 33, 43]
Family support	4	[27, 30, 37, 39]
Family monitoring	2	[36, 37]
Peer pressure	7	[2–30, 35, 36, 40, 42]
Having a boyfriend/girlfriend	8	[5, 25, 27, 28, 35, 36, 40, 41]
Parental occupation	2	[5, 24]
Pornography exposure	14	[5, 24, 26, 28, 29, 31, 32], 34– [36, 42]
Substance/Alcohol use	9	[5, 24, 25], 27– [29, 31, 32, 42]

The classification of adolescent stages followed WHO and UNFPA definitions

This review identified nine key factors influencing adolescents’ tendency to engage in premarital sexual activity: knowledge [31, 32, 36, 38–40], religion [1, 5, 28, 31, 33, 43], family support [27, 30, 37, 39], exposure to pornography [5, 24, 26, 28, 29, 31, 32, 34–36, 42], peer pressure [2–30, 35, 36, 40, 42], dating relationships (having a boyfriend or girlfriend) [5, 25, 27, 28, 35, 36, 40, 41], parental occupation [5, 24], substance/alcohol use [5, 24, 25, 27–29, 31, 32, 42], and family monitoring [36, 37]. Of these nine factors, exposure to pornography, particularly pornography, was the most reported and had a significant influence on premarital sex among the adolescents.

### Factors influencing premarital sex among adolescents based on the theory of planned behavior

**Fig. 2:**
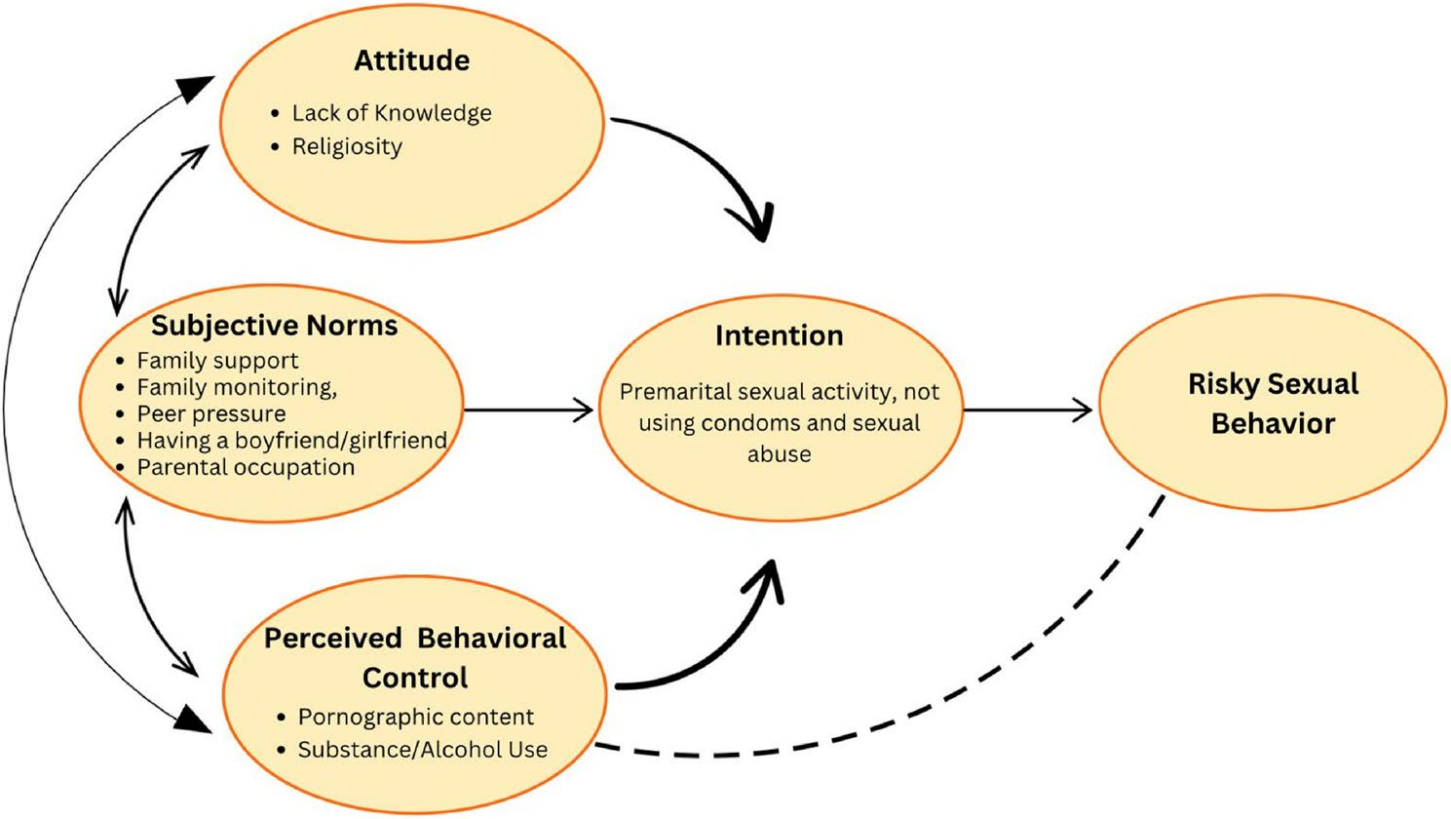
Model of factors related to premarital sexual behavior among adolescents in conservative societies based on the Theory of Planned Behavior (TPB)

Based on the findings of this review, nine factors were frequently associated with premarital sexual relationships among the adolescents. The TPB explains that a person’s intention and behavior to act are influenced by three main factors: attitude toward the behavior, subjective norms, and subjective norms, which together form the intention to engage in premarital sexual relations [45]. The nine factors influencing premarital sex in this study were categorized into three domains based on the theory of planned behavior (Fig. 2).

#### Attitude toward behavior

This category explains how individuals view behavior either positively or negatively [45]. Two factors were included in the attitudes toward behavior between knowledge and religion. Lack of knowledge was significantly associated with premarital sex in adolescents; six studies showed a strong relationship between knowledge and premarital sex, and adolescents who had sufficient knowledge about the impact of premarital sex tended to avoid premarital sex [31, 32, 36, 38–40]. Six studies showed that religiosity had a significant effect on premarital sex in adolescents. Adolescents who are less involved in religious activities and who have a decreased belief in maintaining bodily purity are more likely to engage in premarital sex [1, 5, 28, 31, 33, 43].

#### Subjective norm

The subjective norm category explains the social influence perceived by individuals from the closest people to perform or avoid a behavior [45]. Five factors have a significant relationship with premarital sex in adolescents: family support, family monitoring, peer pressure, having a boyfriend/girlfriend, and parents’ occupation. Four studies emphasized the importance of family support in reducing the risk of premarital sexual behavior among adolescents [27, 30, 37, 39]. Two studies have shown that adolescents with good family monitoring tend to have better moral values and are less likely to engage in premarital sex [36, 37]. Seven studies revealed that peer pressure can make premarital sexual behavior seem more normal, as well as a lack of comprehensive information regarding the impact of premarital sex on adolescents [2–30, 35, 36, 40, 42]. Eight studies found that adolescents who have boyfriends/girlfriends feel more pressured to engage in premarital sex [5, 25, 27, 28, 35, 36, 40, 41]. Two other studies reported a significant relationship between parental occupation and premarital sexual behavior in adolescents [5, 24].

#### Perceived behavioral control

This category describes the extent to which adolescent behavior is influenced or controlled by external factors. Two factors are included in this domain: exposure to pornographic media and substance/alcohol use. More than half of the studies included in this review reported a significant relationship between pornographic content and premarital sex among adolescents. Exposure to pornographic content is one of the most influential external factors for adolescents, and it was the most frequently reported factor in this review, with 14 studies reporting a significant relationship [5, 24, 26, 28, 29, 31, 32, 34–36, 42]. Additionally, the substance/alcohol use factor indicates that adolescents who consume substances or alcohol may lose the ability to make decisions and control themselves, ultimately leading them to engage in premarital sexual activity, as reported in nine studies [5, 24, 25, 27–29, 31, 32, 42].

## Discussion

Our review identified 23 studies that addressed premarital sex among adolescents in conservative societies. These studies integrated evidence related to factors influencing adolescent premarital sexual behavior using the Theory of Planned Behavior (TPB). Our findings confirm that attitudes, subjective norms, and perceived behavioral control play a significant role in shaping adolescents’ intentions and behaviors, which is consistent with the TPB framework.

### Sexual norms and gender differences in Conservative societies

Conservative societies tend to have strict sexual norms, in which premarital sex is considered taboo and forbidden. In many countries such as Africa and Asia, premarital sex is often considered a sensitive issue. Discussions about sex are often seen as supporting such behavior, which can encourage teenagers to engage in premarital sex. In Africa, social norms show differences in freedom of speech about sex, particularly between men and women. Men are freer to discuss this topic, while women are often restricted or even prohibited from talking about premarital sex. Data from the analyzed study also showed that premarital sexual initiation was more common in men. In Africa, the prevalence of premarital sex is 39.4%, whereas in Southeast Asia, it is only 4.2%.

This finding indicates that men are freer to explore their sexuality, while women are more influenced by stricter social norms. In Southeast Asia, cultural values and social norms are heavily influenced by the religions that prohibit adultery. Cultural pressure to maintain purity, dignity, self-esteem, and honor is highly valued as it is considered a reflection of one’s morality. Additionally, the social and legal sanctions imposed on premarital sexual behavior further reinforce this view, influencing adolescents’ perspectives on premarital sex as unacceptable. Interestingly, despite the high value placed on religious principles in this conservative society, this often contrasts with the higher prevalence of premarital sex among male adolescents, even though social pressure and religious norms are very strong. This indicates that, although conservative social norms persist, factors such as media influence, friendships, and access to sexual information still affect teenagers’ decisions to engage in premarital sex. This difference confirms that, although conservative norms are similar in various places, culture and location influence how these norms are applied to men and women.

### The factors associated premarital sex in among adolescents

Knowledge and religiosity emerged as consistent protective factors, with religiosity consistently functioning as a protective factor against risky sexual behavior during adolescence, although its influence weakened in early adulthood [46]. Adolescents with adequate reproductive health knowledge were less likely to engage in premarital sex [31, 32, 36, 38–40], whereas lower religiosity was associated with higher risk [1, 5, 28, 31, 33, 43]. These findings are consistent with those of previous research by Koletić et al. (2021), which showed that religious beliefs and comprehensive sexual health knowledge can delay sexual initiation and reduce risky sexual practices [47]. Therefore, knowledge and religiosity can promote negative attitudes toward premarital sex and encourage healthier behavioral choices. Similar to the patterns observed in conservative societies in Asia, religiosity shapes moral boundaries that discourage premarital sexual relations, while knowledge of sexual health alone does not guarantee a protective effect. This indicates that religiosity functions more as a normative deterrent, while knowledge plays a complementary role, but is insufficient unless supported by strong social and cultural norms.

The normative influence was also proven to be highly significant. Family involvement in supporting [27, 30, 36, 37, 39] and parental monitoring [36, 37] of children is protective, whereas parental employment is indirectly correlated with family supervision and support for children. Peer pressure [2–30, 35–37, 40] and romantic relationships [5, 27, 28, 35, 36, 40] increase the likelihood of premarital sex. This finding highlights the dual nature of social norms: While family and cultural or religious expectations generally discourage premarital sexual relationships, peer and dating relationships often exert pressure in the opposite direction. Such findings are consistent with the social influence theory [48], which emphasizes the power of perceived expectations from those closest to us during adolescence.

Perceived behavioral control, which is most consistently influenced by exposure to pornographic content [5, 24, 26, 28, 29, 31, 32, 34–36, 42]. and substance/alcohol use [5, 24, 25, 27–29, 31, 32, 42] can collectively weaken self-control in adolescents and strengthen their intention to engage in premarital sexual activity, especially in contexts where sex education is limited. Other factors that undermine behavioral control include low self-efficacy, poor negotiation skills [31, 32, 36, 40], and restricted condom availability [24, 39]. Collectively, these findings highlight how external and individual control factors may either constrain or enable adolescents to manage their sexual behavior.

The interaction between attitudes, subjective norms, and perceived behavioral control is important in influencing premarital sexual behavior among adolescents in conservative societies. Adolescents’ attitudes toward premarital sex are influenced by their knowledge and religiosity, which in turn are influenced by the social norms around them (family, peers, and partners). Additionally, external factors, such as media exposure and alcohol consumption, can influence the extent to which they feel that they have control over premarital sexual behavior. Next, the intention to engage in premarital sex was influenced by attitudes, subjective norms, and perceived behavioral control. This desire reflects the tendency of adolescents to engage in premarital sex, often without considering health risks, such as sexually transmitted diseases or unwanted pregnancies. This leads to the emergence of risky sexual behaviors such as premarital sex, not using condoms, and sexual abuse, which are consequences of intentions influenced by attitudes, subjective norms, and perceived behavioral control.

Recent evidence indicates that digital and social media platforms have expanded the way adolescents are exposed to sexual content and engage in risky sexual practice. Social media platforms are increasingly used not only as sources of sexual content but also as spaces that facilitate behaviors, such as seeking sexual partners or engaging in transactional sex, thereby further weakening adolescents’ perceived control over their sexual behavior [49]. Specifically, digital platforms have become the primary channel for adolescents to access sexual content, influencing their perceived behavioral control and involvement in premarital sexual relationships. Evidence from Ethiopia indicates that the use of social media such as Facebook and Twitter is significantly associated with premarital sexual behavior [29]. Similarly, internet use and mobile phone ownership increase the likelihood of premarital sexual relations through easier access to sexual material [24]. Other studies from Sub-Saharan Africa and Asia have consistently identified exposure to pornographic content as a strong predictor of premarital sex [5, 39, 40].

### Strengths and limitations of review

This study is grounded in the Theory of Planned Behavior (TPB) framework, providing a strong theoretical foundation and ensuring conceptual clarity in understanding premarital sexual behavior among adolescents. By focusing on studies conducted in conservative societies, this review offers specific insights relevant to contexts in which cultural and religious norms strongly influence adolescent sexual health behavior. Alignment with widely recognized behavioral theories also strengthens the validity of thematic analysis.

However, this study had several limitations. First, the use of less-specific keywords during the literature search may have resulted in the inclusion of irrelevant articles and potential omission of relevant studies. Second, since most of the included studies were conducted in developing countries with conservative social norms, the findings of this review cannot be generalized to all adolescent populations, particularly those living in societies with more permissive attitudes toward premarital sexual behavior. The results are most applicable to adolescents in conservative societies and may not reflect the factors influencing risky sexual behavior among adolescents in different cultural or social settings. Another limitation of this review was the heterogeneity of the study design and measurement tools across the included studies. Variations in sampling strategies, definitions of premarital sex, and tools used to assess associated factors may have influenced the comparability of findings and consistency of interpretations. Additionally, possible publication bias should be acknowledged as studies reporting significant associations were more likely to be published and included. Furthermore, variations in how “marital sex” was defined across cultural contexts may have influenced the interpretation and comparability of the findings among different studies. As a result, the synthesis presented here should be viewed as mapping broad patterns, rather than providing precise pooled estimates.

Third, most of the included studies employed cross-sectional designs, which restrict the ability to establish causal relationships; longitudinal or experimental evidence was largely absent. Fourth, although the review focused on conservative settings, important within-country variations, such as the differences between tribal and urban adolescents, were not adequately captured. Fifth, subgroup analyses, particularly gender-based differences and the experiences of underrepresented populations, such as LGBTQ + youth, have rarely been reported, limiting the generalizability of the findings. Finally, while pornography exposure has consistently been identified as a determinant of premarital sexual behavior, the influence of contemporary digital media, including social media, dating applications, and influencer culture, remains underexplored. Another limitation of this review was that only English-language studies were included, which may have led to the exclusion of relevant evidence published in other languages.

### Implications to policy and future research

This review’s findings underscore various avenues for addressing policies and practices in conservative societies. Implementing a school-based reproductive health curriculum, recommended by the WHO and UNESCO [50], and peer education and mentoring programs, which have proven effective in the Southeast Asian context, offers an additional way to counter the negative influence of peers, as well as implementing the curriculum to increase adolescent knowledge and shape healthier attitudes toward premarital sex in conservative regions [51].

The implementation of this intervention must prioritize cultural and religious factors within the community. Sexuality education can be integrated into the existing school curriculum through value-based and belief-aligned modules and delivered collaboratively by teachers, school counselors, and religious educators to ensure that the content remains acceptable to local norms. Digital literacy programs should be integrated into character education or ICT subjects, with a focus on the critical evaluation of online content, as well as technical support from the Ministry of Education and youth organizations. Family based interventions can be implemented through community workshops and religious meetings, empowering parents to openly communicate about sexuality within an acceptable moral framework. Collaboration between religious figures and community health workers is crucial for increasing credibility and acceptance within the community.

Additionally, digital literacy programs and family interventions are recommended for communities, as integrating digital literacy into school- and community-based reproductive health is increasingly relevant to reducing the influence of pornography and promoting safer online behavior. Family based interventions, including community-based family interventions (such as parental monitoring and communication training), can reinforce protective cultural and religious values, whereas active community involvement ensures that programs remain culturally acceptable. Additionally, community- and faith-based campaigns tailored to local norms offer a promising strategy for strengthening protective behaviors while reducing the stigma surrounding reproductive health [50, 51].

Although this research has provided useful insights into the factors influencing premarital sexual behavior, several research gaps need to be addressed. Existing studies have largely focused on urban adolescents, with limited representation of rural adolescents or specific subpopulations, such as LGBTQ + youth. Further research is needed to explore the influence of socioeconomic factors, local cultural norms, and access to information on sexual education across diverse social environments. Additionally, despite extensive research in South Asia and Africa, few studies have explored the influence of social and religious norms in Southeast Asia or the Middle East, which have distinct cultural and religious dynamics. Longitudinal studies are crucial for tracking how these factors change over time and how social norms and religious values influence adolescents’ decisions in the long run.

## Conclusion

This scoping review synthesizes factors influencing premarital sexual behavior among adolescents in conservative societies using the Theory of Planned Behavior (TPB) framework. The review identified nine key factors associated with premarital sexual behavior, categorized into three domains based on TPB: attitudes, subjective norms, and perceived behavioral control. Adolescents’ attitudes toward premarital sex are influenced by religiosity and knowledge, with those with greater knowledge and stronger religious beliefs being less likely to engage in premarital sex. Subjective norms play a significant role in shaping adolescents' intentions and behaviors, influenced by family support, family monitoring, peer pressure, having a boyfriend/girfriend and parental occupation. Perceived behavioral control is strongly influenced by factors such as exposure to pornography and substance use, which weaken adolescents’ ability to control their sexual behavior, especially in contexts where sexual health education is limited.

The findings of this review highlight the complex interplay between social norms, individual attitudes, and external control factors in determining adolescent engagement in premarital sexual activities. This study emphasizes the importance of addressing all three domains in interventions aimed at reducing risky sexual behaviors in conservative societies. Future research should explore differences between subgroups, such as those based on gender and LGBTQ + population, as well as longitudinal studies to track behavioral changes over time.

## Supplementary Information

Supplementary Material 1.

Supplementary Material 2.

Supplementary Material 3.

## Data Availability

Not applicable.
