# Supplementary material for: A scoping review of factors associated with premarital sex-related risky sexual health behavior among adolescents in conservative societies based on the theory of planned behavior
Source: BMC Public Health. 2025 Dec 4;26:127. doi: 10.1186/s12889-025-25665-x (PMC12797830; doi:10.1186/s12889-025-25665-x)
Supplement: Supplementary file 1 — Supplementary Material 1. [file 12889_2025_25665_MOESM1_ESM.docx]

**Supplementary file 2. Quality appraisal**

| **Study** | **Study Design** | **JBI** |
| --- | --- | --- |
| [21] | Cross-Sectional | 75% (6/8) |
| [22] | Cross-Sectional | 87.5% (7/8) |
| [23] | Cross-Sectional | 87.5% (7/8) |
| [37] | Cross-Sectional | 75% (6/8) |
| [24] | Cross-Sectional | 100% (8/8) |
| [38] | Cross-Sectional | 87.5% (7/8) |
| [25] | Cross-Sectional | 100% (8/8) |
| [26] | Cross-Sectional | 75% (6/8) |
| [35] | Cross-Sectional | 87.5% (7/8) |
| [31] | Cross-Sectional | 100% (8/8) |
| [32] | Cross-Sectional | 100% (8/8) |
| [36] | Cross-Sectional | 87.5% (7/8) |
| [39] | Cross-Sectional | 87.5% (7/8) |
| [33] | Cross-Sectional | 87.5% (7/8) |
| [40] | Cross-Sectional | 100% (8/8) |
| [27] | Cross-Sectional | 100% (8/8) |
| [28] | Cross-Sectional | 100% (8/8) |
| [34] | Cross-Sectional | 75% (6/8) |
| [29] | Cross-Sectional | 75% (6/8) |
| [30] | Cross-Sectional | 100% (8/8) |
