# Supplementary material for: A scoping review of factors associated with premarital sex-related risky sexual health behavior among adolescents in conservative societies based on the theory of planned behavior
Source: BMC Public Health. 2025 Dec 4;26:127. doi: 10.1186/s12889-025-25665-x (PMC12797830; doi:10.1186/s12889-025-25665-x)
Supplement: Supplementary file 2 — Supplementary Material 2. [file 12889_2025_25665_MOESM2_ESM.docx]

**Supplementary file 1. search strategy**

| **Database** | **Keywords** |
| --- | --- |
| PubMed | (("Adolescent"[MeSH Terms] OR Adolescents [text word] OR Teen [text word] OR Teens) AND ("premarital sex"[MeSH Terms] OR premarital sexual intercourse)) AND (predictor [MeSH Terms] OR factors [text word] OR determinant) |
| Scopus | ( TITLE-ABS-KEY ( adolescent OR adolescents OR teen OR teens ) ) AND ( TITLE-ABS-KEY ( "Premarital sex" OR "Premarital sexual intercourse" ) ) AND ( TITLE-ABS-KEY ( "predictor" OR "factors" OR "determinant" ) ) |
| EBSCOhost | "Adolescent" OR "Adolescents" OR "Teen" OR "Teens" AND "Premarital sex" OR "Premarital sexual intercourse" AND "Predictor" OR "Factors" OR "Determinant" |
| Cochrane | Adolescent OR Adolescents OR Teen OR Teens OR Youth OR Young AND premarital sex OR premarital sexual intercourse OR premarital sexual behaviour |
