# Supplementary material for: A scoping review of factors associated with premarital sex-related risky sexual health behavior among adolescents in conservative societies based on the theory of planned behavior
Source: BMC Public Health. 2025 Dec 4;26:127. doi: 10.1186/s12889-025-25665-x (PMC12797830; doi:10.1186/s12889-025-25665-x)
Supplement: Supplementary file 3 — Supplementary Material 3. [file 12889_2025_25665_MOESM3_ESM.pdf]

Table. Data extraction

Author (Reference)	Study Design					Premarital sex	
	Design	Country	Sample size	Gender	Age (Adolescent Stage)	Prevalence	Predictors
Shitu et al. 2023 [24]	Quantitative Cross-Sectional	Ethiopia	7389	Male 3500 (47.4%)<br>Female 3889 (52.6%)	19–24 years (Late/Youth)	10.8%	• Internet use<br>• Occupation<br>• Alcohol use<br>• Khat chewed
Biratu el al. 2022 [25]	Quantitative Cross-Sectional	Ethiopia	312	Male 170 (54.5%)	15–19 years (Middle-Late)	24.4%	• Having a boyfriend/girlfriend<br>• Substance use/alcohol use
Bogale et al. 2014 [26]	Mixed-method Cross-Sectional	Ethiopia	826	Male 401 (48.5%)<br>Female 425 (51.5%)	15–24 years (Middle-Late/Youth)	19%	• Watching pornographic movies<br>• Living with friends<br>• Discuss sexuality with close friends
Manaf et al. 2014 [5]	Quantitative Cross-Sectional	Malaysia	1328	Male (44%)<br>Female (55%)	< 18 years (Late/Youth)	5.4%	• Religion<br>• Occupation<br>• Have lover<br>• Reading or watching porn<br>• Drink alcohol
Teffera et al. 2015 [27]	Quantitative Cross-Sectional	Ethiopia	302	Male (73.5%)<br>Female (26.5%)	15–24 years (Middle-Late/Youth)	42.7%	• Having a boyfriend/girlfriend<br>• Smoke tobacco/alcohol<br>• Discuss sexuality and RH issues with family/relatives

Author (Reference)	Study Design					Premarital sex	
	Design	Country	Sample size	Gender	Age (Adolescent Stage)	Prevalence	Predictors
Ahanhanzo et al. 2018 [40]	Quantitative Cross-Sectional	Benin	360	Male (54.67%)<br><br>Female 545.33%	10-24 years (Early-Late/Youth)	31.66%	• Father's level of education<br>• Communication between parents and teenagers<br>• Opinion about premarital sex<br>• Exposure to pornographic movies
WL et al. 2019 [28]	Quantitative Cross-Sectional	Ethiopia	380	Male 222 (46.2%)<br><br>Female 258 (53.8%)	15-24 years (Middle-Late/Youth)	22.5%	• Attending religious education<br>• Having a boyfriend/girlfriend<br>• Pornography watching<br>• Drink alcohol
Behulu et al. 2019 [29]	Quantitative Cross-Sectional	Ethiopia	600	Male 296 (49.3%)<br><br>Female 304 (50.7%)	15-18 years (Middle-Late)	31.3%	• Discussing sexual issues with close friends<br>• Peer pressure
Hayee et al. 2021 [38]	Quantitative Cross-Sectional	Thailand	700	Male 230<br><br>Female 470	14-19 years (Middle-Late)	9.0%	• Parent-adolescent communication<br>• Parental monitoring<br>• Perceived peer norms
Pradanie et al. 2022 [34]	Quantitative Cross-Sectional	Indonesia	109	Male 86 (78.9%)<br><br>Female 23 (21.1%)	15-18 years (Middle-Late)	18.6%	• Religiosity
Wulandari et al. 2024 [35]	Quantitative Cross-Sectional	Indonesia	530	Male 117 (22.1%)	< 19 years (Late/Youth)	4.2%	• Exposure to pornography

Author (Reference)	Study Design					Premarital sex	
	Design	Country	Sample size	Gender	Age (Adolescent Stage)	Prevalence	Predictors
				Female 413 (77.9%)			
Sangkaew et al. 2023 [39]	Quantitative Cross-Sectional	Thailand	674	Male 243 (36.1%)<br><br>Female 431 (63.9%)	13-19 years (Early-Late)	69%	• Educational status<br>• Acceptance of sex behavior
Paudel et al. 2023 [41]	Quantitative Cross-Sectional	Nepal	415	Male 194 (46.7%)<br><br>Female 221 (53.3%)	> 18 years (Late/Youth)	21.2%	• Father's education<br>• Ever been in a relationship<br>• Has a close friend who has experienced premarital sex<br>• Exposure to pornography
Yudanagara et al. 2024 [36]	Quantitative Cross-Sectional	Indonesia	8876	Male (100%)	15-24 years (Middle-Late/Youth	14.7%	• Access to media<br>• Currently having a girlfriend<br>• Have friends who have ever had sex before marriage<br>• Ever advised/influenced by a friend/someone to have sexual intercourse
Budu et al. 2023 [42]	Quantitative Cross-Sectional	Africa	87.924	Female (100%)	15-24 years (Middle-Late/Youth)	39.4%	• Religion<br>• Media exposure
Mulugeta et al. 2014 [30]	Quantitative Cross-Sectional	Ethiopia	1093	Female (100%)	16-24 years (Middle-Late/Youth)	30.8%	• Watching pornography<br>• Peer pressure<br>• Chewing khat

Author (Reference)	Study Design					Premarital sex	
	Design	Country	Sample size	Gender	Age (Adolescent Stage)	Prevalence	Predictors
Salih et al. 2015 [31]	Quantitative Cross-Sectional	Ethiopia	624	Female (100%)	14-20 years Middle-Late/Youth)	29.3%	• Discussed sexual activity-related issues with parents<br>• Elder sister who had started sexual activity before marriage<br>• Close friends who had started sexual activity before marriage<br>• Peer Pressure
Ismainar et al. 2017 [37]	Quantitative Cross-Sectional	Indonesia	400	*Unclear*	13-14 years (Early-Middle)	53.5%	• Courtship status<br>• Knowledge<br>• Pornography access<br>• Harmonious family<br>• Peers<br>• Parents monitoring
Meleko et al. 2017 [32]	Quantitative Cross-Sectional	Ethiopia	320	Male 173 (57.3%)<br><br>Female 129 (42.7%)	15-24 years (Middle-Late/Youth)	25.2%	• Mother's educational status<br>• Attended religious services<br>• Khat chewing<br>• Cigarette smoking<br>• Drinking alcohol<br>• Watching pornography<br>• Peer pressure
Regassa et al. 2016 [33]	Quantitative Cross-Sectional	Ethiopia	704	Male 529 (75.1%)<br><br>Female 175 (24.9%)	15-19 years (Middle-Late)	28.4%	• Using social media<br>• Chewing khat<br>• Having comprehensive knowledge of HIV<br>• Watching pornographic films<br>• Alcohol use
Cao et al. 2015 [43]	Quantitative Cross-Sectional	China	4.769	Female (100%)	18-22 years (Late/Youth)	18.1%	• Contraceptive behavior<br>• Having multiple sex partners

Author (Reference)	Study Design					Premarital sex	
	Design	Country	Sample size	Gender	Age (Adolescent Stage)	Prevalence	Predictors
Wong et al. 2009 [44]	Quantitative Case-control	Singapore	1.044	*Unclear*	14-19 years (Middle-Late)	36.4%	• Alcohol use<br>• Drug use<br>• Peer pressure<br>• Media exposure<br>• History of Sexual abuse
Kupoluyi et al. 2025 [45]	Quantitative Cross-Sectional	Nigeria	23,446	Female (100%)	15-24 years (Middle-Late/Youth)	31.3%	• Religion<br>• Media exposure<br>• Community

Note: The classification of adolescent stages followed WHO and UNFPA definitions (10-13 early, 14-16 middle, 17-19 late adolescence, and 20-24 youth).
